# Experiences with telemedicine among family medicine residents at king saud university medical city during the COVID-19 pandemic: a cross-sectional study

**DOI:** 10.1186/s12909-023-04295-0

**Published:** 2023-05-05

**Authors:** Ibrahim AlFawaz, Abdullah A. Alrasheed

**Affiliations:** Department of Family and Community Medicine, College of Medicine, King Saud University, Riyadh, 11362 Saudi Arabia

**Keywords:** Telemedicine, Covid-19, Residency training, Medical Education, Saudi Arabia

## Abstract

**Background:**

The healthcare system experienced various challenges during the coronavirus disease 2019 (COVID-19) pandemic, and a wide range of safety measures were implemented, including limiting the number of patients allowed to visit primary care clinics and follow-up through telemedicine clinics. These changes have accelerated the growth of telemedicine in medical education and affected the training of family medicine residents throughout Saudi Arabia. Therefore, this study aimed to evaluate the experiences of family medicine residents with telemedicine clinics as a part of their clinical training during the COVID-19 pandemic.

**Methods:**

A cross-sectional study was conducted with 60 family medicine residents at King Saud University Medical City, Riyadh, Saudi Arabia. An anonymous 20-item survey was administered between March and April 2022.

**Results:**

The participants included 30 junior and 30 senior residents, with a 100% response rate. The results revealed that most (71.7%) participants preferred in-person visits during residency training, and only 10% preferred telemedicine. In addition, 76.7% of the residents accepted the inclusion of telemedicine clinics in training if such clinics constituted not more than 25% of the training program. Moreover, most participants reported receiving less clinical experience, less supervision, and less discussion time with the attending supervisor when training in telemedicine clinics compared with in-person visits. However, most (68.3%) participants gained communication skills through telemedicine.

**Conclusions:**

Implementing telemedicine in residency training can create various challenges in education and influence clinical training through less experience and less clinical interaction with patients if it is not structured well. With the growth of digital healthcare, further structuring and testing of a paradigm that involves using telemedicine in residents’ training programs prior to implementation should be considered for better training and patient care.

## Background

Telemedicine has existed since the early 1960s. Over the years, it has developed and spread considerably, becoming a widely used tool for patient care. The World Health Organization defined telemedicine as “the delivery of health care services, where distance is a critical factor, by all health care professionals using information and communication technologies” [1 p. 9]. Consulting through telephone consultation provides a promising alternative to in-person visits for general practice care [2]. Moreover, telemedicine reduces inefficiencies in the delivery of healthcare, such as reducing patient travel and waiting time [3]. In addition, telemedicine was found to be effective in specialty consultations, primary care assessments, preoperative assessments, and postoperative follow-ups [4, 5].

The World Health Organization declared a global pandemic after cases of coronavirus disease 2019 (COVID-19) were confirmed worldwide [6]. During the COVID-19 pandemic, many hospitals in Saudi Arabia reduced the number of patients allowed to visit primary care clinics by more than 75% of their maximum capacity. This reduction led to the implementation of telemedicine clinics as a part of patient care. Telemedicine is an attractive solution to minimize the risk of virus transmission [7].

The use of telemedicine is growing in clinical practice, and medical residents of the current generation grew up using technology as a major part of their daily lives. Medical residents believe that interactions with telemedicine during their training serve as an important educational tool that supports their understanding of core competencies in practice-based learning, medical knowledge, and patient care [7]. In a 2015 survey of 207 family medicine residencies nationwide, the majority of program directors reported that their facilities had some form of telehealth services; however, actual use was limited and infrequent [8]. The COVID-19 pandemic led the Ministry of Health of Saudi Arabia to accelerate the growth of digital health by creating and developing mobile health applications and adopting telemedicine in primary care clinics of many tertiary hospitals to improve patient care and minimize the risk of infection. Since many of the tertiary hospitals are teaching hospitals, the adoption of telemedicine introduced a new set of challenges to the family medicine residency program that family medicine residents are involved in as a part of their training. To the best of our knowledge, only one study, conducted in the USA, assessed the perceptions of family medicine residents regarding the use of telemedicine in their training [9]. However, the sample of that study was small. Therefore, the present study aimed to assess experiences with telemedicine among family medicine residents during the COVID-19 pandemic.

## Methods

### Study group

A cross-sectional study was conducted with family medicine residents at King Saud University Medical City, Riyadh, Saudi Arabia. The family medicine residency program had 60 residents; all of them were included in the study, with a 100% response rate. Telemedicine clinic visits were introduced to the residents in October 2020. Prior to the clinic appointment, family medicine consultants sorted the booked cases based on the patient’s reason for booking and used their judgment to decide whether the patient required an in-person or telemedicine consultation. The cases included new and follow-up visits. Telemedicine clinics were conducted primarily through telephone rather than video calls. To assess the residents’ experience, a 20-item questionnaire survey was administered between March and April 2022. The questionnaire was anonymous to ensure confidentiality.

### Questionnaire design

A validated electronic questionnaire from a similar study conducted in the USA was adapted after obtaining the author’s permission [10]. A few adjustments were made to the questionnaire to match the family medicine program. The adjustments were reviewed and approved by three research experts of family medicine consultants. Questions 1 and 2 related to demographics, including gender and post-graduation year level (residency level). Questions 5, 6, 7, 16, and 17 focused on providing patient-centered care. Question 8 and its subsections assessed residents’ confidence. Common diseases managed in family clinics, including diabetes, dyslipidemia, hypertension, hypothyroidism, osteoarthritis, depression and anxiety, chronic headaches, urinary tract infections, back pain, and congestive heart failure, were chosen. Questions 9–11 evaluated system-based practice and work in interdisciplinary teams. Questions 12–15 focused on practice-based learning and improvement and asked family medicine residents about their clinical experience and level of supervision through telemedicine clinics. Questions 18–20 assessed the influence of experiences with telemedicine clinics on family medicine residents’ career plans and inquired their opinions on the acceptable ratio of telemedicine in residency training.

### Statistical analysis

The data were analyzed using SPSS v. 23.0. Chi-square and Fisher’s exact tests were used to analyze the difference (1) between post-graduation levels (juniors and seniors) and practice-based learning parameters (amount of attending supervision, clinical experience gain, and acceptable percentage of telemedicine practice) and (2) between post-graduation levels and other variables including communication skill gain, preference of telemedicine practice, and effect of telemedicine on future career decision. P < 0.05 was considered indicative of a statistically significant difference. In addition, Spearman’s correlation was used to test the association between post-graduation level and residents’ confidence.

## Results

The questionnaire was distributed to 60 residents (30 juniors and 30 seniors), with a 100% response rate. Of the participants, 48.3% (29) were men and 51.7% (31) were women (Table 1). Prior to the COVID-19 pandemic, none of the residents had telemedicine experience. The results indicated that if a patient did not answer the call, 81.7% (49) of the participants attempted to call 2–3 times, while only 18.3% (11) attempted to call 4 times or more. Most (47; 78.3%) of the participants could handle 6–8 telemedicine visits per clinic (Table 1).

**Table 1:** Participant demographics, practice characteristics, and career choice (n = 60)

	Percentage	Number
**Gender**		
Male	48.3%	29
Female	51.7%	31
**PGY Level**		
PGY 1	25.0%	15
PGY 2	25.0%	15
PGY 3	25.0%	15
PGY 4	25.0%	15
**Number of phone call attempts per patient visit**		
1	0.0%	0
2	10.0%	6
3	71.7%	43
4 or more	18.3%	11
**Number of telemedicine appointments per clinic**		
1–3	1.7%	1
4–5	20.0%	12
6–8	78.3%	47
**Preference regarding practice in residency training**		
In-person visit	71.7%	43
Telemedicine	10.0%	6
No preference	18.3%	11
**Your telemedicine experience affects your future career decision**		
Yes	53.3%	32
No	11.7%	7
No difference	35.0%	21
**Acceptance of practicing telemedicine in your residency program**		
0%	8.3%	5
25%	76.7%	46
50%	15.0%	9
75%	0.0%	0
100%	0.0%	0

**Fig. 1:**
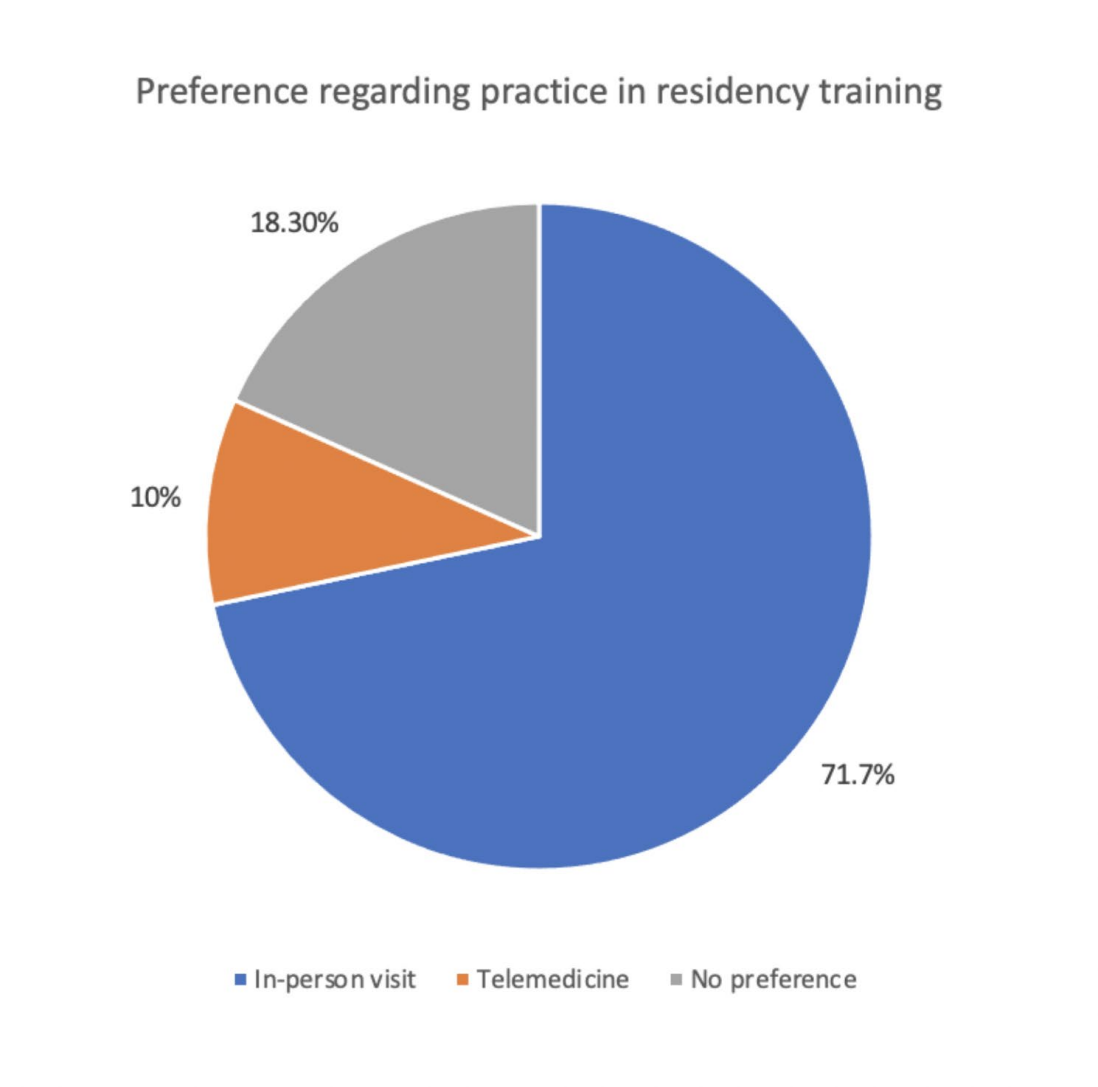
Preference regarding practice in residency training

An overall 53.3% (32) of the participants thought that telemedicine may affect their future career decision. However, in-person visit practice was the preferred mode in residency training among most (43; 71.7%) of the participants, and only 10% (6) preferred telemedicine. In addition, 76.7% (46) of the participants accepted the implementation of telemedicine clinics as a part of their training as long as they constituted not more than 25% of the training program (Table 1)(Fig. 1).

**Fig. 2:**
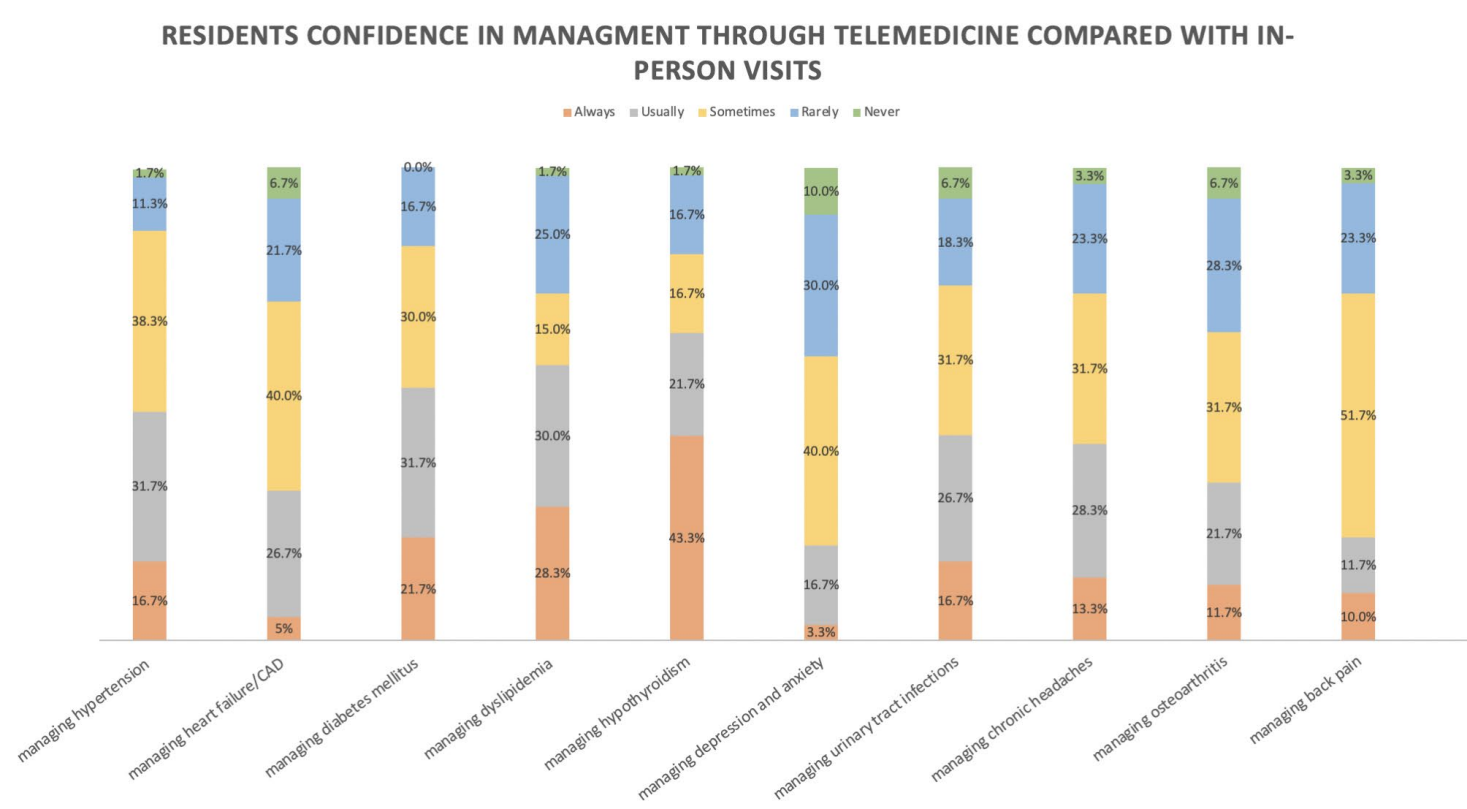
Residents confidence in management through telemedicine compared with in-person visits

**Fig. 3:**
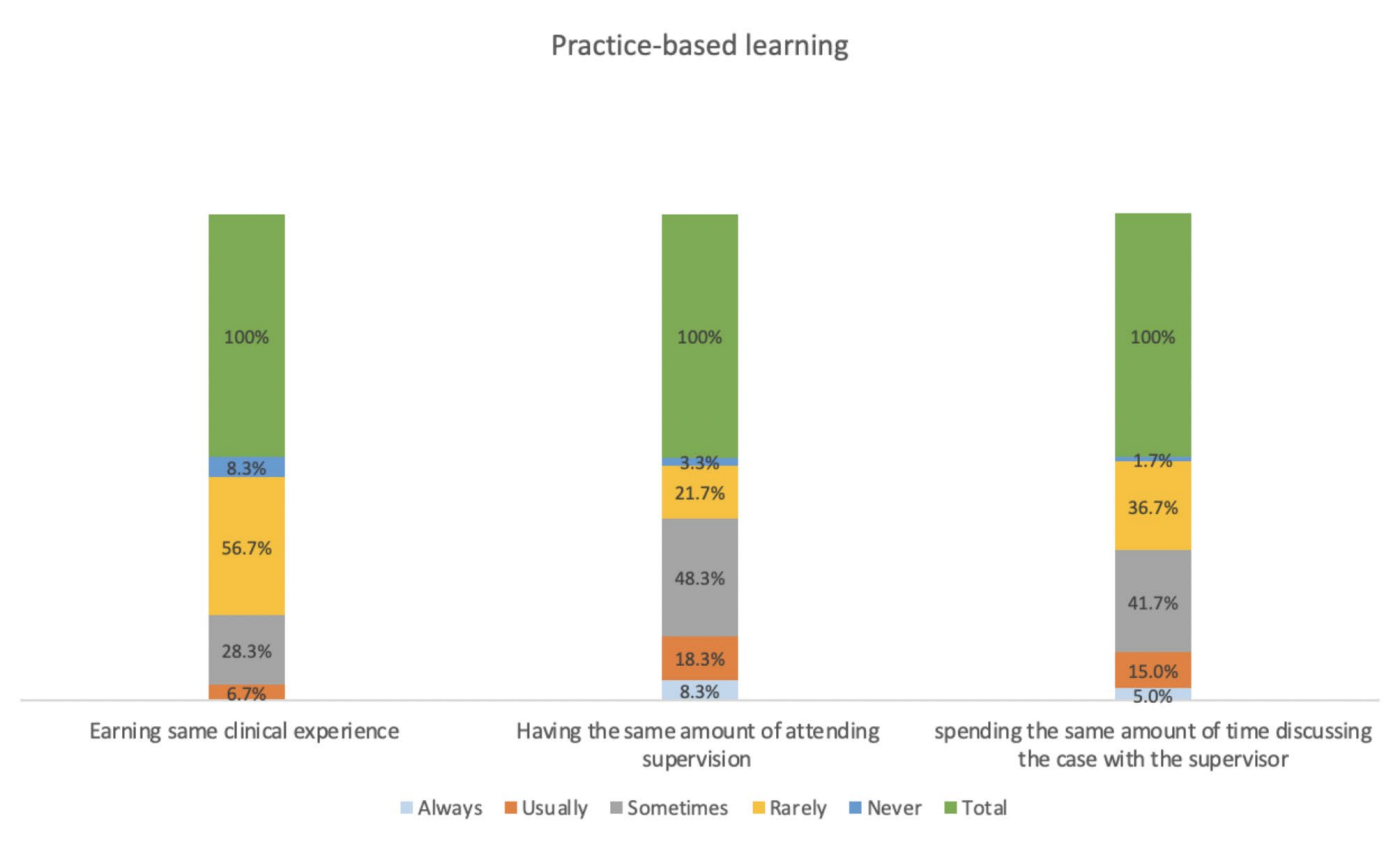
Practice-based learning

Compared with in-person visits, most participants reported receiving less clinical experience, less supervision, and less discussion time with the attending supervisor when training in telemedicine clinics. However, most (41; 68.3%) participants gained communication skills through telemedicine (Table 2). The participants were mostly confident in managing chronic conditions, such as hypertension, dyslipidemia, diabetes mellitus, and hypothyroidism, through telemedicine. In contrast, most participants were not confident in dealing with back pain through telemedicine. The participants stated that telemedicine does not provide the same quality of care as in-person visits because of an increased number of patients lost to follow-up, significant language barriers, and patients feeling uncomfortable with discussing their complaints through telemedicine (Table 2)(Figs. 2 and 3).

**Table 2:** Resident satisfaction (n = 60)

Practice-based learning	Always	Usually	Sometimes	Rarely	Never
Do you feel that you earn the same clinical experience through telemedicine compared with in-person visit?	0.0% (0)	6.7% (34)	28.3% (17)	56.7% (34)	8.3% (5)
Do you feel that you have the same amount of attending supervision during telemedicine compared with in-person visits during the COVID-19 pandemic?	8.3% (5)	18.3% (11)	48.3% (29)	21.7% (13)	3.3% (2)
Do you feel that during attending supervising you spend the same amount of time discussing the case during telemedicine compared with in-person visits during the COVID-19 pandemic?	5.0% (3)	15.0% (9)	41.7% (25)	36.7% (22)	1.7% (1)
**Resident confidence**					
Do you feel confident managing hypertension through telemedicine compared with in-person visits?	16.7% (10)	31.7% (19)	38.3% (23)	11.7% (7)	1.7% (1)
Do you feel confident managing heart failure/CAD through telemedicine compared with in-person visits?	5.0% (3)	26.7% (16)	40.0% (24)	21.7% (13)	6.7% (4)
Do you feel confident managing diabetes mellitus through telemedicine compared with in-person visits?	21.7% (13)	31.7% (19)	30.0% (18)	16.7% (10)	0.0% (0)
Do you feel confident managing dyslipidemia through telemedicine compared with in-person visits?	28.3% (17)	30.0% (18)	15.0% (9)	25.0% (15)	1.7% (1)
Do you feel confident managing hypothyroidism through telemedicine compared with in-person visits?	43.3% (26)	21.7% (13)	16.7% (10)	16.7% (10)	1.7% (1)
Do you feel confident managing depression and anxiety through telemedicine compared with in-person visits?	3.3% (2)	16.7% (10)	40.0% (24)	30.0% (18)	10.0% (6)
Do you feel confident managing urinary tract infections through telemedicine compared with in-person visits?	16.7% (10)	26.7% (16)	31.7% (19)	18.3% (11)	6.7% (4)
Do you feel confident managing chronic headaches through telemedicine compared with in-person visits?	13.3% (8)	28.3% (17)	31.7% (19)	23.3% (14)	3.3% (2)
Do you feel confident managing osteoarthritis through telemedicine compared with in-person visits?	11.7% (7)	21.7% 13)	31.7% (19)	28.3% (17)	6.7% (4)
Do you feel confident managing back pain through telemedicine compared with in-person visits?	10.0% (6)	11.7% (7)	51.7% (31)	23.3% (14)	3.3% (2)
**System-based practice in interdisciplinary teams**					
Do your telemedicine patients visit the hospital to get blood work (labs) done less often than in-person patients?	6.7% (4)	20.0% (12)	45.0% (27)	20.0% (12)	8.3% (5)
Do your telemedicine patients visit the hospital for imaging (mammogram, X-ray, CT scan, MRI scan, etc.) less often than in-person patients?	1.7% (1)	15.0% (9)	55.0% (33)	25.0% (15)	3.3% (2)
**Patient-centered care from the residents’ standpoint**					
Have you ever experienced your patient not picking up the telephone?	6.7% (4)	28.3% (17)	56.7% (34)	8.3% (5)	0.0% (0)
Do you feel difficulty conducting medication reconciliation through telemedicine compared with in-person visits?	10.0% (6)	15.0% (9)	36.7% (22)	26.7% (16)	11.7% (7)
Do you feel that telemedicine causes a larger language barrier between you and your patient (even with a phone interpreter) compared with in-person clinic visits?	11.7% (7)	21.7% (13)	31.7% (19)	25.0% (15)	10.0% (6)
Does telemedicine increase the number of patients lost to follow-up?	5.0% (3)	35.0% (21)	28.3% (17)	23.3% (14)	8.3% (5)
Do you feel that patients are not comfortable discussing their medical conditions with you via telephone?	1.7% (1)	10.0% (6)	41.7% (25)	38.3% (23)	8.3% (5)
Do you think that patients receive the same level of care during telemedicine compared with in-person visits?	0.0% (0)	26.7% (16)	45.0% 27)	25.0% (15)	3.3% (2)

Fisher’s exact test and chi-square test were conducted to investigate the association between post-graduation levels (juniors and seniors) and practice-based learning parameters (amount of attending supervision, clinical experience gain, and acceptable percentage of telemedicine practice). There were no statistically significant associations (p = 0.37, p = 0.42, and p = 0.47, respectively; Table 3). In addition, the same tests were used to evaluate the relationship between post-graduation levels and other variables, including communication skill gain, preference of telemedicine practice, and effect of telemedicine on their future career decision. Similarly, there were no significant statistical associations (p = 0.78, p = 1.00, and p = 0.61, respectively; Table 4). Spearman’s correlation was used to test the association between post-graduation level and resident confidence variables. The results revealed a statistically significant correlation between post-graduation level and treating hypothyroidism and urinary tract infections (p = 0.01, and p = 0.01, respectively; Tables 4 and 5).

**Table 3:** Association between post-graduation level and practice-based learning parameters

			PGY Level		Total	Chi-square test p-value
			Senior	Junior		
Attending supervision	>or = 50%	Count	21	9	30	0.371
		Expected count	22.5	7.5	30	
	< 50%	Count	24	6	30	
		Expected count	22.5	7.5	30	
Clinical experience	>or = 50%	Count	9	12	21	0.417
		Expected count	10.5	10.5	21	
	< 50%	Count	21	18	39	
		Expected count	19.5	19.5	39	
Telemedicine practice percentage	>or = 50%	Count	3	6	9	0.472
		Expected count	4.5	4.5	9	
	< 50%	Count	27	24	51	
		Expected count	25.5	25.5	51	

**Table 4:** Association between post-graduation level and communication skill gain, preference of telemedicine practice, and effect of telemedicine on future career decision

			PGY Level		Total	Chi-square test p-value
			Senior	Junior		
Communication skill gain	YES	Count	21	20	41	0.781
		Expected count	20.5	20.5	41	
	NO	Count	9	10	19	
		Expected count	9.5	9.5	19	
Telemedicine visit preference	YES	Count	3	3	6	1.000
		Expected count	3	3	6	
	NO	Count	27	27	54	
		Expected count	27	27	54	
Telemedicine affects career decision	YES	Count	17	15	32	0.605
		Expected count	16.0	16.0	32	
	NO	Count	13	15	28	
		Expected count	14	14	28	

**Table 5:** Association between post-graduation level and residents’ confidence variables

	PGYLevel	Spearman’s correlation p-value
Hypertension	N (60)	0.747
Heart Failure	N (60)	0.539
Dyslipidemia	N (60)	0.107
Diabetes mellitus	N (60)	0.879
Hypothyroidism	N (60)	0.014
Depression and anxiety	N (60)	0.602
UTI	N (60)	0.01
Chronic headache	N (60)	0.237
Osteoarthritis	N (60)	0.844
Back pain	N (60)	0.611

Correlation is significant at the 0.05 level (2-tailed)

## Discussion

Residency training for all specialties was affected during the COVID-19 pandemic, leading to the use of technology as a part of medical education and training. During the COVID-19 pandemic, various fields of the healthcare system, such as telemedicine, medical education, and patient care, were affected [11–13].

Our results demonstrated that most of the family medicine residents surveyed in this study preferred in-person visits rather than implementing telemedicine in their training program. In addition, our findings indicated that most participants felt confident in making decisions for managing chronic conditions. Apart from this is because acute conditions such as new onset proctological complaints require in-person visits for further physical examination [20]. Therefore, through our observation, in terms of decision making, we believe that telemedicine should be implemented in residency training regardless of seniority level. Moreover, most residents stated that telemedicine increased the number of patients who did not complete the required labs and imaging studies or were lost to follow-up. In addition, many participants reported experiencing a language barrier caused by telemedicine. These problems can affect the continuity of care and patient trust in their physicians, which are core principles of family medicine practice. Nevertheless, most participants reported gaining communication skills while using telemedicine, which is another core principle of family medicine.

Most of our findings were consistent with that of the Lincoln Medical Center study [9]. Most participants reported gaining less clinical experience through telemedicine clinics and preferred in-person visits, and the majority of the participants reported that the inclusion of telemedicine might affect their future career decision. The participants stated that they prefer only 25% or less of their training to include telemedicine. This could be due to the lower amount of supervision and discussion time with the attending supervisor during the clinic. The reduced supervision and discussion time, in turn, could be due to the COVID-19 pandemic and the high number of attempted calls from the residents to the patients, leading to fewer patients answering and decreased time for discussion with the supervisor. The high number of telemedicine appointments per clinic could be another possible explanation. Previous research conducted in other developed countries identified the absence of a policy framework for telemedicine as a key factor that influenced the implementation and sustainability of telemedicine [14, 15]. One study showed that most residents expressed an overall concern regarding their overall preparedness to conduct telemedicine visits and their ability to provide high-quality care to their patients [16]. The increased use of technology in medical education and training necessitates an organized policy and regulations for implementing telemedicine clinics, including requiring the attendant physician to physically supervise trainees and discuss cases with them. This could improve training for use of telemedicine. A study conducted in France reported that most residents acknowledged the growth and expansion of telemedicine, reported not being well trained to use telemedicine, and were aware that training is mandatory to provide high-quality care [17]. Training in using telemedicine could increase the confidence level of residents during clinical practice [9]. The acceptance of telemedicine practice in Saudi Arabia could result in its significant growth [18]. Appropriate training in telemedicine could alleviate disparities in care access in rural and urban areas [19]. Telemedicine plays an important role in digital health growth. Residents are a part of healthcare providers, and proper training during residency programs can help develop telemedicine.

## Conclusion

Residents need to advance their clinical skills and judgment throughout their training, as this will make them better physicians and allow them to approach and effectively manage patients remotely and provide high-quality care. Implementing telemedicine in residency training and medical education requires clear protocols and paradigms designed and tested by experts that help achieve optimal training and enhance patient care.

Our study’s strength lies in being the first study in the Middle East with the largest sample size. However, this study had several limitations. First, it was a single-center study with a small sample involving only the department of family medicine; therefore, it may not represent the residents in other specialties or other hospitals. Second, the study regarded only phone consultations; thus, other modalities of telemedicine should be considered in the future. Third, this study was conducted during the COVID-19 pandemic, and future normalized circumstances should be considered. Finally, the future involvement of telemedicine in residency training requires follow-up studies to assess its effect on such training.

## Data Availability

The datasets used and/or analysed during the current study are available from the corresponding author on reasonable request.

## Abbreviations

COVID-19: coronavirus disease 2019
